# Coming home to die? the association between migration and mortality in rural South Africa

**DOI:** 10.1186/1471-2458-9-193

**Published:** 2009-06-18

**Authors:** Paul Welaga, Victoria Hosegood, Renay Weiner, Caterina Hill, Kobus Herbst, Marie-Louise Newell

**Affiliations:** 1 Navrongo Health Research Centre, Navrongo, Ghana; 2 Centre for Population Studies, London School of Hygiene and Tropical Medicine, London, UK; 3 Africa Centre for Health and Population Studies, University of KwaZulu-Nata, Mtubatuba, South Africa; 4 School of Public Health, University of Witwatersrand, Johannesburg, South Africa; 5 Centre for Paediatric Epidemiology, Institute of Child Health, University College London, London, UK

## Abstract

**Background:**

Studies on migration often ignore the health and social impact of migrants returning to their rural communities. Several studies have shown migrants to be particularly susceptible to HIV infection. This paper investigates whether migrants to rural households have a higher risk of dying, especially from HIV, than non-migrants.

**Methods:**

Using data from a large and ongoing Demographic Surveillance System, 41,517 adults, enumerated in bi-annual rounds between 2001 and 2005, and aged 18 to 60 years were categorized into four groups: external in-migrants, internal migrants, out-migrants and residents. The risk of dying by migration status was quantified by Cox proportional hazard regression. In a sub-group analysis of 1212 deaths which occurred in 2000 – 2001 and for which cause of death information was available, the relationship between migration status and dying from AIDS was examined in logistic regression.

**Results:**

In all, 618 deaths were recorded among 7,867 external in-migrants, 255 among 4,403 internal migrants, 310 among 11,476 out-migrants and 1900 deaths were registered among 17,771 residents. External in-migrants were 28% more likely to die than residents [adjusted Hazard Ratio (aHR) = 1.28, P < 0.001, 95% Confidence Interval (CI) (1.16, 1.41)]. In the sub-group analysis, the odds of dying from AIDS was 1.79 [adjusted Odd ratio (aOR) = 1.79, P = 0.009, 95% CI (1.15, 2.78)] for external in-migrants compared to residents; there was no statistically significant difference in AIDS mortality between residents and out-migrants, [aOR = 1.25, P = 0.533, 95% CI (0.62–2.53)]. Independently, females were more likely to die from AIDS than males [aOR = 2.35, P < 0.001, 95% CI (1.79, 3.08)].

**Conclusion:**

External in-migrants have a higher risk of dying, especially from HIV related causes, than residents, and in areas with substantial migration this needs to be taken into account in evaluating mortality statistics and planning health care services.

## Background

South Africa has a high level of circular migration with people migrating into urban areas primarily to look for jobs whilst maintaining contact with their family members in rural areas [1,2]. Several studies have shown the existence of an association between migration and the spread of HIV, with migrants being particularly susceptible to HIV infection [3,4]. In an early study in KwaZulu-Natal, people who had recently migrated or changed their places of residence were three times more likely to be infected with HIV than residents [5]. A subsequent study on HIV-1 concordance and discordance among migrant and non-migrant couples in South Africa showed that the direction of spread is not only from returning migrant men to their rural partners, but also from resident women to their migrant partners [4]. Therefore both migrants and residents in rural areas are vulnerable to HIV.

Studies on migration often ignore the health and social impact of migrants returning to their rural communities. Migrants continue to maintain links with their households in rural areas [5,6] and there is some evidence to suggest that migrants move back to their rural households for care and support when seriously ill [7]. This phenomenon has been amplified by the emergence of AIDS. A study in Thailand revealed that the most common place for HIV infected adults to spend the terminal stage of the illness was in the parental home and the most common caregiver at this stage was a parent, usually a mother [8]. A study on households' experiences of HIV and AIDS in the study area showed evidence of return migration of household members working elsewhere if they became ill and stopped working [9]. However, many rural areas, particularly in sub-Saharan Africa, lack adequate health facilities and personnel to cater for the sick in these places. Therefore, returning migrants to these rural communities could influence the burden of disease and mortality rates locally. This would have implications for health delivery systems in rural areas, particularly in areas or countries with high prevalence of HIV. South Africa has witnessed rapid growth in the spread of HIV. It has the highest number of people living with HIV, and AIDS is the leading cause of death in the country [10,11].

This paper quantifies the overall mortality differentials between migrants and non-migrants in a rural community in South Africa and investigates more specifically whether returning migrants have a higher probability of dying from AIDS than non-migrants.

## Methods

### The study area

The study area is situated in the south-eastern part of the Hlabisa sub-district in Umkhanyakude, northern KwaZulu Natal, South Africa [12] about 220 km north of the provincial capital of Durban. Health services include a district hospital and a network of 6 primary health care clinics and two mobile clinic teams [[10], ]. In the study area, the crude HIV incidence rate per 100 person-years between 2003 and 2005 was 3.8 [95% confidence interval (CI), 3.2–4.6] in women aged 15–49 years and 2.3 (95% CI, 1.8–3.1) in men aged 15–54 years [13]. Overall, 21.5% of residents between the ages of 15–49 in the area were HIV-infected, highest among women aged 25–29 years and men aged 35–39 years [14].

### The Demographic Surveillance System (DSS)

Since 2000, a demographic surveillance system known as the Africa Centre Demographic Information Systems (ACDIS) has been in place in the study site [[12], ]. The ACDIS maps approximately 11,000 inhabited homesteads in a 435 square kilometre area, with a total population of approximately 90,000 [[12], ]. Field workers visit all households in the area twice a year and longitudinal, socio-economic and demographic information, including the residence and survival status of all household members is updated. The residential status in ACDIS is in two groups (residents and non-residents), where "residents," are defined as "individuals who report keeping their belongings and spending most nights at the surveyed household," and "non-residents" defined as "individuals whose residence is elsewhere but maintain connections with the household through periodic visits" [15,16]. Approximately 30% of all household members are non-resident.

### Data

Verbal autopsy is routinely conducted on every death notified in the Demographic Surveillance Area. Verbal autopsy is an epidemiological tool that is used to assess cause of death in settings where hospital based records are lacking. Trained nurses carry out structured interviews with close relatives or the main caregiver of the person before they died, which is then analysed by clinicians to give a cause of death [10]. For the purpose of this study which was carried out in 2006 as part of a master's course in population-based field epidemiology, verbal autopsy data for 2001 and 2002 was available at the time of the analysis and therefore, the cause of death analysis was limited to this dataset.

For the overall mortality rates, the data included members of homesteads aged 18 to 60 years in the Demographic Surveillance Area between 1^st ^January 2001 and 31^st ^December 2005. The data was limited to the age group of 18 to 60 years because this group of people were more likely to be involved in migration .

### Statistical Analysis

To determine the overall mortality differentials, household members were divided into four groups: external in-migrants, internal migrants, out-migrants and residents. People in the Demographic Surveillance Area (DSA) who were resident on 1^st ^January 2001 and remained resident until they either died or were censored at the last visit date of the fieldworker in 2005 were classified as resident individuals. People who migrated from outside the DSA into the study area after 1^st ^January 2001 and either died or were still resident in the area at the last visit date of the fieldworker in 2005 were classified as external in-migrants. Those who changed residency within the study area after 1^st ^January 2001 were considered as internal migrants. Out-migrants were people who moved out of the study area during the study period.

To assess the association between migrating into the area and subsequent mortality, the last migration event that occurred between 1^st ^January 2001 and 31^st ^December 2005 was used. The total person time contribution for each participant was calculated as the number of days they had been observed as members of their households starting from the day they entered that migration status in the DSA between 1^st ^January 2001 and 31^st ^December 2005 using their most recent residency episode. The membership episode for people who did not die during the period was right censored on 31^st ^December 2005 or the date their membership ended whichever was earliest. Where an individual had more than one residency episode, the most recent was chosen. Whilst this excluded some residency episodes that did not end with a death, the number of such individuals was small. The person years for in-migrants starts from the day they last moved into residency in the area. Household members who were not in the DSA at any time between 1st January 2001 and 31st December 2005 were not included in the analyses.

### Cause of death analysis

All deaths which occurred in 2001 and 2002 were classified into four groups, external in-migrant deaths, internal migrant deaths, resident deaths and out-migrant deaths based on the most recent migration episode before their death and where they died. Deaths that occurred outside the study area were classified as out-migrant deaths.

To assess the risk of dying from AIDS related complications among the various groups, a binary variable was generated that took the value 1 if an individual was reported to have died from AIDS and 0 if not. Both univariate and multivariate logistic regression models were used to determine the odds of dying from AIDS.

### Overall mortality analysis

Cox proportional hazard regression technique was used to quantify the risk of dying for residents, external in-migrants and internal migrants. The Cox proportional hazard assumptions were tested and found to be not violated [17,18]. Potential confounders such as age, socio-economic status using number of household assets [19] and level of education were controlled for in the multivariate model. The household assets considered were bicycle, block-maker, car, electric stove with oven, electric hot plate, electric kettle, fridge/freezer, gas cooker, bednet, lorry/tractor, motorcycle/scooter, radio, car battery, bed, sofa/sofa suit, sewing machine, table/chair, telephone, cell-phone, television set, video cassette recorder and wheelbarrow. Educational level was grouped into four based on the grade an individual had completed (Table 1). Mortality rates per 1000 person years were obtained by Kaplan Meier survival analysis [20].

**Table 1:** Characteristics of participants in the overall survival analysis

Variable	Total N = 41,517	External in-migrants n = 7,867	Internal migrants n = 4,403	Out-migrants 11,476	Residents n = 17,771	P-value
**Age (years)**						
Median	28	28	28	24	33	
Internal Quartile	22 – 39	23 – 36	22 – 37	21–31	22 – 45	
Range						
**Sex**						
Female	23,372 (56.3%)	4,371 (55.6%)	2,804 (63.7%)	5,865 (51.1%)	10,332 (58.1%)	<0.001
Male	18,145 (43.7%)	3,496 (44.4%)	1,599 (36.3%)	5,611 (48.9%)	7,439 (41.9%)	
						
**Education**						
						
Grade 1 to 4	6,790 (16.4%)	1,018 (12.9%)	670 (15.2%)	1,036 (9.0%)	4,066 (22.9%)	
Grade 5 to 12	26,733 (64.4%)	5,001 (63.6%)	2,803 (63.7)	7,309 (63.7%)	11,620 (65.4%)	
Tertiary	275 (0.7%)	15 (0.2%)	14 (0.3%)	112 (1.0%)	134 (0.8%)	<0.001
Don't know	2,295 (5.5%)	478 (6.1%)	290 (6.6%)	605 (5.3%)	922 (5.2%)	
Missing	5,424 (13.1%)	1,355 (17.2%)	626 (14.2%)	2,414 (21.0%)	1,029 (5.8%)	
						
**Age category**:						
18 to 25	17,484 (42.1%)	3,051 (38.8%)	1,801 (40.9%)	6,384 (55.6%)	6,248 (35.2%)	
26 to 40	14,380 (34.6%)	3,410 (43.4%)	1,767 (40.1%)	3,910 (34.1%)	5,293 (29.8%)	<0.001
41 to 60	9,653 (23.3%)	1,406 (17.9%)	835 (19.0%)	1,182 (10.3%)	6,230 (35.1%)	
						
**Economic status**						
< 7 assets	18,735 (45.1%)	3,605 (45.8%)	2,312 (52.5%)	4,605 (40.1%)	8,213 (44.2%)	
>= 7 assets	19,744 (47.6%)	3,755 (47.7%)	1,670 (37.9%)	5,413 (47.2%)	8,906 (50.1%	<0.001
Missing	3,038 (7.3%)	507 (6.4%)	421 (9.6%)	1,458 (12.7%)	652 (3.7)%	
						
**Mortality rate/1000 person years**	**22.5**	**42.0**	**26.5**	**8.6**	**20.2**	

P-value measures the association between each of the variables and migration status

95% confidence intervals for rates, hazard ratios and odd ratios were estimated. Tests of statistical significance for differences between rates and determining significant associations between variables were based on the chi-square test [21]. Analysis was done using STATA 10 [22].

Ethics approval for the study was obtained from the Human Research Ethics Committee of University of the Witwatersrand. Ethics approval for the Africa Centre Demographic Information System was obtained from the University of KwaZulu-Natal Nelson R. Mandela School Of Medicine.

## Results

### Overall mortality over five years

Table 1 gives the background characteristics of participants included in the survival analysis who were ever resident household members of the cohort between 1^st ^January 2001 and 31^st ^December 2005. In the overall mortality analysis, 3,083 deaths were reported of a total of 41,517 people between 18 and 60 years of age, 618 deaths among 7,867 external in-migrants, 255 among 4,403 internal migrants, 310 among 11,476 out-migrants and 1900 deaths were registered among 17,771 residents. The median age of participants was 28 years with the majority (42.1%) aged 18 to 25 years. The overall mortality rate over five years was 22.5 per 1000 person years. External in-migrants had the highest mortality rate, followed by internal migrants, residents and out-migrants (Table 2). In univariate analysis, a person who moved into the area during this period was 1.60 times more likely to die than someone who was resident, and after adjusting for other factors, the risk was 1.28. There was no significant difference in the risk of dying between internal migrants and residents. Out-migrants were 70% less likely to die than residents. Males were 1.36 times more likely to die during the follow-up period of five years than females.

**Table 2:** Relative risk* for univariate and multivariate models for the 5 years survival experience by migration status

	Univariate model		Multivariate Model	
Variable	n	HR [95%CI]	Adjusted HR [95% CI]	P-Value

**Sex**				
Females	23,372	1	1	
Male	18,145	1.17 [1.09–1.26]	1.36 [1.27–1.46]	<0.001

**Migration status**				
External in-migrants	7,867	1.60 [1.45–1.75]	1.28 [1.16–1.41]	<0.001
Internal migrants	4,403	1.01 [0.89–1.16]	0.89 [0.78–1.02]	0.102
Out-migrants	11,476	0.34 [0.30–0.38]	0.30 [0.27–0.34]	<0.001
residents	17,771	1	1	

**Education**				
Grade 1 to 4	6,790	1	1	
Grade 5 to 12	26,733	0.44 [0.40–0.48]	0.74 [0.67–0.81]	<0.001
Tertiary	275	1.38 [1.00–1.93]	1.80 [1.29–2.53]	0.001
Don't know	2,295	0.38 [0.30–0.47]	0.45 [0.36–0.55]	<0.001
Missing	5,424	3.32 [3.01–3.67]	5.09 [4.54–5.72]	<0.001

**Age category**:				
18 to 25	17,484	1	1	
26 to 40	14,380	4.60 [4.13–5.11]	3.55 [3.18–3.95]	<0.001
41 to 60	9,653	4.24 [3.80–4.74]	3.28 [2.91–3.70]	<0.001

**Economic status**				
>= 7 assets	18,735	1	1	
< 7 assets	19,744	1.25 [1.16–1.35]	1.32 [1.22–1.43]	<0.001
Missing	3,038	3.33 [2.94–3.77]	1.19 [1.04–1.37]	0.010

* Hazard ratio (HR) with 95% confidence intervals (95%CI).

Variables controlled for were sex, migration status, educational level, age and socioeconomic status.

### Cause of death

Table 3 gives the characteristics of participants included in the cause of death analysis. The median age of the 1212 people who died between 1^st ^January 2001 and 31^st ^December 2002 was 35 years (range 18 to 60). Overall, 795 (65.6%) of all deaths were due to AIDS, about 74% of all female deaths and 57% of all male deaths. There were 116 (15.0%) external in-migrants who died of HIV related causes, 32 internal migrants (4.0%), 63 (7.7%) out-migrants and 586 (73.7%) were resident individuals. The proportion of AIDS deaths for external in-migrants and internal migrants was higher than among residents and out-migrants.

**Table 3:** Characteristics of participants included in the cause of death analysis

**Variable**	**Total no. (%)**	**AIDS deaths (%)**	**Other deaths (%)**	**P-value**
**Sex**				
Male	599 (49.4)	340 (56.8)	259 (43.2)	<0.001
Female	613 (50.6)	455 (74.2)	158 (25.8)	

**Migration status**				
External in-migrants	148 (12.2)	116 (78.4)	32 (21.6)	
Internal migrants	44 (3.6)	32 (72.7)	12 (27.3)	0.003
Out-migrants	97 (8.0)	61 (62.9)	36 (37.1)	
residents	923 (76.2)	586 (63.5)	337 (36.5)	

**Education**				
Grade 1 to 4	301 (24.8)	178 (59.1)	123 (40.1)	
Grade 5 to 12	412 (34.0)	282 (68.5)	130 (31.5)	0.005
Tertiary	19 (1.6)	8 (42.1)	11 (57.9)	
Missing	480 (39.6)	327 (68.1)	153 (31.9)	

**Age category**:				
18 to 25	162 (13.4)	88 (54.3)	74 (45.7)	
26 to 40	612 (50.5)	468 (76.5)	144 (23.5)	<0.001
41 to 60	438 (36.1)	239 (54.6)	199 (45.4)	

**Economic status**				
< 7 household assets	563 (46.5)	377 (67.0)	186 (33.0)	
>= 7 household assets	433 (35.7)	266 (61.4)	167 (38.6)	0.050
Missing	216 (17.8)	152 (70.4)	64 (29.6)	

**Total**	1,212	795 (65.6)	417 (34.4)	

P-value measures the association between the variables and cause of death

In this sub-group, external in-migrants were 79% more likely to die from AIDS than residents (Table 4). However, although internal migrants had a higher risk of dying from AIDS compared to residents, this was not statistically significant. Out-migrants were 9% less likely to die from AIDS than residents; females were 2.35 times more likely to die from AIDS than males and people aged 26 to 40 years were 3.47 times more likely to die from AIDS compared to those aged 18 to 25 years. Members in households with less than 7 assets were 40% more likely to die from AIDS than those with 7 or more assets.

**Table 4:** Odds ratio (OR) estimates and 95% confidence intervals for factors associated with the likelihood of dying with AIDS, 2001–2002

	Univariate (Unadjusted)				Multivariate (adjusted)		
Variable	number	Odds Ratio	95% CI	P-value	Adjusted Odds Ratio	95% CI	P-value

**Sex**							
Males	599	1	-	-	1	-	-
Females	613	2.19	(1.72, 2.80)	<0.001	2.35	(1.79, 3.08)	<0.001

**Migration status**							
External in-migrants	148	2.08	(1.38, 3.15)	0.001	1.79	(1.16,2.78)	0.009
Internal migrants	44	1.53	(0.78, 3.02)	0.216	1.25	(0.62, 2.53)	0.533
Out-migrants	97	0.97	(0.63, 1.50)	0.907	0.91	(0.58, 1.45)	0.705
Residents	923	1	-	-	1	-	-

**Education**							
Grade 1 to 4	301	1	-	-	1	-	-
Grade 5 to 12	412	1.50	(1.10, 2.04)	0.010	1.40	(0.99, 1.96)	0.057
Tertiary	19	0.50	(0.20, 1.29)	0.151	0.32	(0.12, 0.86)	0.024
Missing	480	1.48	(1.09, 1.99)	0.011	1.21	(0.87, 1.70)	0.258

**Age category**:							
18 to 25	162	1	-	-	1	-	-
26 to 40	612	2.73	(1.90, 3.92)	<0.001	3.47	(2.36, 5.11)	<0.001
41 to 60	438	1.01	(0.70, 1.45)	0.957	1.45	(0.97, 2.17)	0.070

**Economic status**							
>= 7 house assets	563	1	-	-	1	-	-
< 7 household assets	433	1.27	(0.98, 1.65)	0.071	1.40	(1.05, 1.86)	0.021
Missing	216	1.49	(1.05, 2.12)	0.025	1.67	(1.15, 2.42)	0.007

Variables controlled for were sex, migration status, educational level, age and socioeconomic status.

## Discussion

External in-migrants were significantly more likely to die than residents, and in the sub-group analysis they were found to be also more likely to die from AIDS. These findings are in line with our hypothesis that migrants into rural areas may come back to rural households when severely ill, and that the higher AIDS mortality is likely to be related to the age group of migrants, with the pattern lagging the peak of HIV incidence ages [13].

The findings of a higher overall mortality among in-migrants is consistent with a similar study conducted in Agincourt DSS on circular labour migration and mortality in Northeast South Africa [2] in which the annual odds of dying from all causes for returned migrants was between 1.1 and 1.9 times higher than for residents and long-term returned migrants. In this study, socioeconomic status, gender and age were all associated with the risk of death, with males at significantly higher risk of dying than females, and people with low socio-economic status at an increased risk of dying compared to higher socio-economic individuals. These findings are in line with those of other studies [23-27].

This study could not ascertain the health conditions of the migrants at the time they moved into the surveillance area, nor their motivation for migration. It is possible that the decision to migrate for some of the people dying shortly afterwards was not connected to ill health, although the higher AIDS mortality in the sub-group for whom cause of death information was available shows that a substantial proportion of deaths were associated with their chronic condition.

Results from the sub-group analysis of data relating to deaths in 2000 and 2001 showing that the risk of AIDS mortality is also increased among external in-migrants is consistent with previous findings of high prevalence of AIDS among migrants and return migration taking place when the severity of illness experienced by persons with AIDS is substantial [8]. After becoming ill, migrants may not be able to obtain the additional financial support or care they need without moving to be with rural household members. There was however no evidence of a significantly increased risk of dying from AIDS between residents and internal migrants. Out-migrants from the study area have a lower risk of dying from AIDS compared to residents, although this did not reach statistical significance, likely due to the lack of statistical power and small number of events.

Females had a higher risk of dying from AIDS compared to males, which is likely to be partly due to the higher prevalence of HIV among females than among males [13,14]. The population-based HIV surveillance in the study area in 2004 showed a prevalence of 27.2% among women aged 15 to 49 years and 13.4% for men aged 15 to 54 years [13,14]. However, although females were more likely to die of AIDS, males had a significantly higher overall mortality rate than females, most likely due to injuries and accidents. Other factors associated with AIDS mortality are socioeconomic status and age. Adults aged 26 to 40 years were most at risk of dying from AIDS, these ages are those of highest HIV prevalence. A study in Uganda found the highest attributable risk of HIV associated deaths to be among persons aged 20–39 years and women [28]. Even though individuals with tertiary education had a significantly higher overall mortality rate than those with grade 1 to 5 education level, they were less likely to die from AIDS. This could most likely be due to the relatively low HIV prevalence rate among people with tertiary education as a study from the same population showed that one additional year of education reduced the hazard of acquiring AIDS by 7% [29].

A possible limitation of the study is the use of verbal autopsy to determine the probable cause of death. Even though a number of studies have found this instrument to have a high sensitivity and specificity and to be able to reasonably determine most causes of deaths [30,31], it is not the gold standard in determining causes of death. A validation of the verbal autopsy data against hospital records in the study area in 2000 found the sensitivity, specificity and positive predictive value of the verbal autopsies for non AIDS deaths of over 90% [10]. For AIDS deaths, the sensitivity, specificity and positive predictive value were 80%, 82% and 85% respectively [10]; however, although this could potentially lead to misclassification of AIDS deaths, this would be unlikely to be differentially so between external in-migrants and the other groups. The presence of missing data on education and socioeconomic status is a possible limitation but this again would be unlikely to differ substantially by migration category.

Our findings suggest that resource-poor rural areas in Sub-Saharan Africa face an increased demand for health services as a consequence of severely ill people in-migrating to the area. Health services thus need to plan for these additional disease burdens posed by migrants moving into rural areas, whist recognising that most will be returning residents rather than people completely new to the area. There are also implications for rural households who may need to respond not only to an additional dependant adult in the household but also the reduction in household income.

## Conclusion

External in-migrants had a higher risk of dying, especially from AIDS, than residents. It is important that in resource-poor settings with high HIV/AIDS prevalence, disease burdens and mortality be identified and quantified in order to put in place effective interventions to better the health conditions of affected populations.

## Competing interests

The authors declare that they have no competing interests.

## Authors' contributions

PW, VH, RW, CH, KB and MLN contributed to the study design, PW extracted and analyzed the data. PW, VH, RW, CH, KB and MLN participated in the interpretation of data and writing of the manuscript. All authors approved the final manuscript.

## Pre-publication history

The pre-publication history for this paper can be accessed here:


